# Image Transport Through Meter-Long Randomly Disordered Silica-Air Optical Fiber

**DOI:** 10.1038/s41598-018-21480-0

**Published:** 2018-02-15

**Authors:** Jian Zhao, Jose Enrique Antonio Lopez, Zheyuan Zhu, Donghui Zheng, Shuo Pang, Rodrigo Amezcua Correa, Axel Schülzgen

**Affiliations:** 1 CREOL, College of Optics and Photonics, University of Central Florida, Orlando, FL 32816 USA; 2 School of Electronic and Optical Engineering, Nanjing University of Science and Technology, Nanjing, Jiangsu 210094 China

## Abstract

We present a randomly disordered silica-air optical fiber featuring a 28.5% air filling fraction in the structured region, and low attenuation below 1 dB per meter at visible wavelengths. The quality of images transported through this fiber is shown to be comparable to, or even better than, that of images sent through commercial multicore imaging fiber. We demonstrate robust high-quality optical image transfer through 90 cm-long fibers with disordered silica-air structure, more than an order of magnitude improvement compared to previous disordered fiber imaging distances. The effects of variations of wavelength and feature size on transported image quality are investigated experimentally.

## Introduction

Transverse Anderson localization (TAL) was first proposed by Abdullaev *et al*. and De Raedt *et al*.^1,2^. The refractive index profile of the optical wave system introduced by De Raedt *et al*. is random in the transverse plane and invariant in the longitudinal direction^2^. This system is similar to a conventional fiber. The optical beam can propagate in the longitudinal direction of this system maintaining a finite beam cross-section due to the localization effect caused by strong random scattering in the transverse plane. The beam localization radius in the transverse plane depends on the refractive index contrast, the materials filling fractions, and the wavelength. The first experimental observation of TAL was demonstrated by Schwartz *et al*. in 2007^3^. Since then, considerable effort has been made to find applications that benefit from TAL in waveguide structures. In 2012, TAL was confirmed in a polymer random optical fiber with refractive index variations Δn of about 0.1 between the two materials inside the random fiber core^4^. Optical image transport using TAL was demonstrated in this type of polymer random fiber in 2014^5^. Image transported through this random fiber exhibited a quality that was at least comparable to images sent through some of the best commercial multicore imaging fibers, indicating great potential for applications such as biological and medical imaging. However, several challenges remain to make random fibers an actual contender for commercial applications. For instance, the small refractive index contrast (~0.1) is a barrier to reducing the beam localization radius, which limits the image resolution. In addition, there is a large signal attenuation in the polymer random fiber limiting the transmission distance to a few centimeters^5^. For practical applications, much longer image transmission distances are typically desired, and therefore fiber materials with low signal attenuation are required. Due to the high refractive index contrast and low loss, glass-air random fibers (GARFs) with about 50% air filling fraction have been proposed as a potential solution^5,6^. Although previous attempts in GARFs were reported^7,8^, no image transport has been demonstrated yet, a fact that was attributed to the very low air filling fraction (<8%) in the first generation GARFs. Increasing the air filling fraction to a high level while keeping a random structure appears to be a technical challenge.

We recently fabricated a GARF with much higher air filling fraction (~26%) and low attenuation, and demonstrated the first image transport through a 4.6 cm-long sample^9^. In this paper, we show high-quality image transport over distances close to 1 meter utilizing new GARFs with high air filling fraction and smaller feature sizes. We show that the quality of the transported image in this new GARFs is comparable to, or even better than, one of the best commercial multicore imaging fibers. Robust image transport through straight and bent GARFs of 90 cm length is demonstrated. To the best of our knowledge, this is the first time high quality image transport through any meter-long segment of disordered fiber has been realized.

## Results and Discussion

### Image transport in GARF

**Figure 1:**
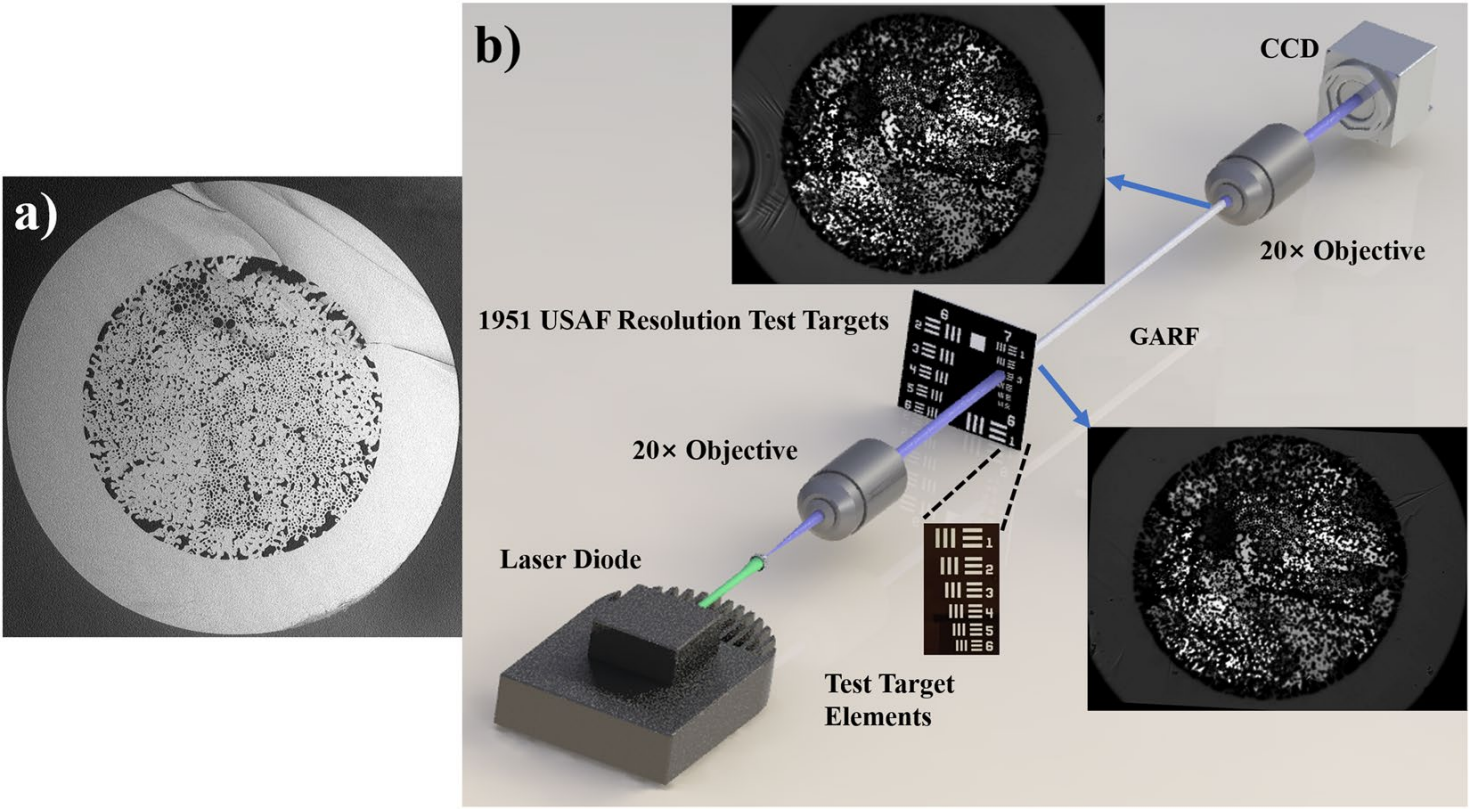
(**a**) SEM cross-section image of GARF(1). (**b**) Schematic of the image transport experimental setup.

Several GARFs have been fabricated at CREOL and, as an example, the cross-section SEM image of GARF(1) is shown in Fig. 1(a). In Fig. 1(b), we also show microscope images of the input and output facets of our 90 cm long GARF(1) sample indicating the invariance of the cross-section along the fiber. The GARFs were fabricated by the stack-and-draw technique. Thousands of silica capillaries with different diameter and air-filling fraction were mixed randomly, and fed into a jacket tube to make preforms. Subsequently, fibers were drawn to desired diameters. The outer diameter of GARF(1) is 414 μm and the diameter of the randomly disordered region is 278 μm with an air filling fraction of about 28.5%. The air-hole areas range from 0.64 μm^2^ to over 100 μm^2^. Statistically, areas of approximately 2.5 μm^2^ (Supplementary Fig. 1(a)) cover the largest area of the randomly disordered region of GARF(1). The attenuation at visible wavelengths measured by the cut-back method was below 1 dB per meter. Here we directly coupled light from single mode fibers into the GARFs. To demonstrate image transmission, we use the experimental setup shown in Fig. 1(b). A 1951 USAF resolution test target (Thorlabs R3L3S1N) is placed directly in front of the cleaved input facet of the fiber under investigation. Both the GARFs and the commercial imaging fibers are cleaved with a large diameter fiber cleaver (Vytran LDC-400). Various elements of the resolution target are illuminated by a collimated beam from a CW laser diode. The light transmitted through the resolution test target is coupled into the disordered fiber region, and the output facet of the fiber is imaged onto a CCD camera (Gentec-EO Beamage-3.0) using a 20x objective.

**Figure 2:**
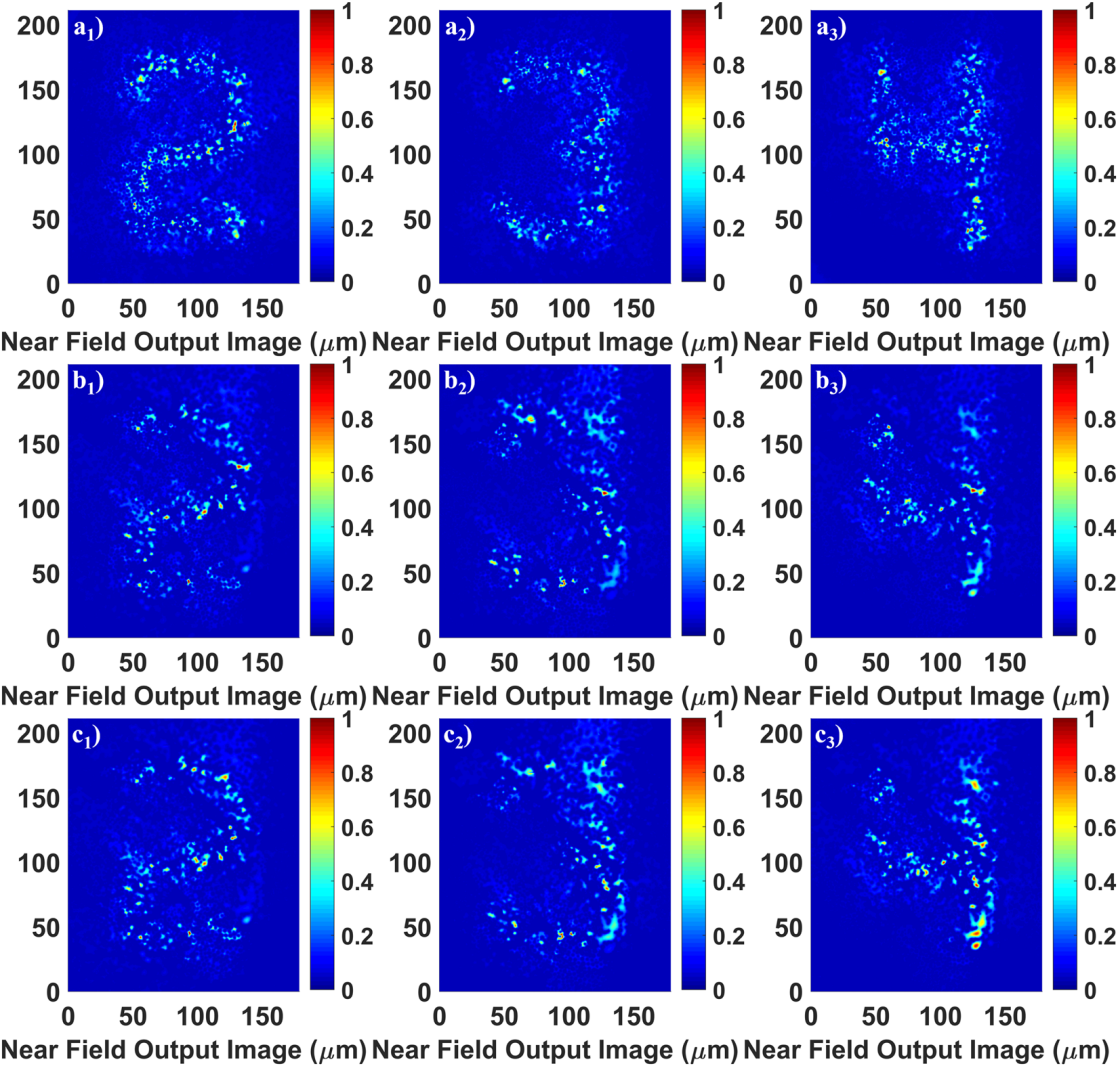
Transported images of different numbers of group 3 on the 1951 USAF test target. (**a1**–**a3**) Show transmission through a 4.5 cm straight GARF(1) sample; (**b1**–**b3**) show transmission through a 90 cm straight GARF(1) sample; (**c1**–**c3**) show transmission through the same 90 cm GARF(1) sample with a 180 degree turn (20 cm bending radius). The wavelength is 405 nm.

To investigate the image transport capability of GARF(1), various numbers from group 3 of the resolution test target are illuminated by 405 nm light. These images are transported through GARF(1) and recorded by the CCD camera. Figure 2 shows transported images of numbers that belong to the same group of the test target and, therefore, have the same sizes of approximately 100 μm × 160 μm. Transported images have been collected for three different cases: (a1–a3) are images after propagation through 4.5 cm of a straight GARF(1) sample, (b1–b3) show images transported through 90 cm of a straight GARF(1), and (c1–c3) show images transported through a 90 cm GARF(1) segment that has a bend to form an 180 degree turn with a bend radius of 20 cm. In all cases, the transmitted image has high visual quality, as well as the same size as the illuminated original target element. All images can be clearly identified even after propagation through 90 cm of GARF(1), and even after bending the 90 cm GARF(1) with a 20 cm radius. Using the same 4.5 cm of straight GARF(1) sample and 405 nm light, we also recorded a real-time dynamic video of the transported image by scanning all numbers from group 3 across the randomly disordered region of GARF(1) (see Supplementary Video 1). The video rate is 30 frames per second, and all the transported images can be identified clearly.

Theoretically, the resolution of the transported images is determined by the transverse localization beam radius and the width of the point spread function of the setup. In practice, the quality of the GARF end cleaving and the variation of fiber dimension and cross-section along the fiber also limit the imaging resolution and quality. Considering the large draw ratio of our GARF, the refractive index profile remains relatively invariant along several meters of the fiber. It is found that this kind of small perturbation does not noticeably disturb the TAL effect^6^. The visual image quality is only slightly lower for transmission through 90 cm of GARF compared to a 4.5 cm segment indicating only slight variations of fiber dimension along the light propagation direction. Extending the imaging fiber length to almost one meter corresponds to an improvement of more than an order of magnitude compared to previous image transportation experiments through disordered fibers^5^. This demonstrates the potential of this new type of imaging fibers for practical applications.

### Comparison with the commercial multicore image fiber

**Figure 3:**
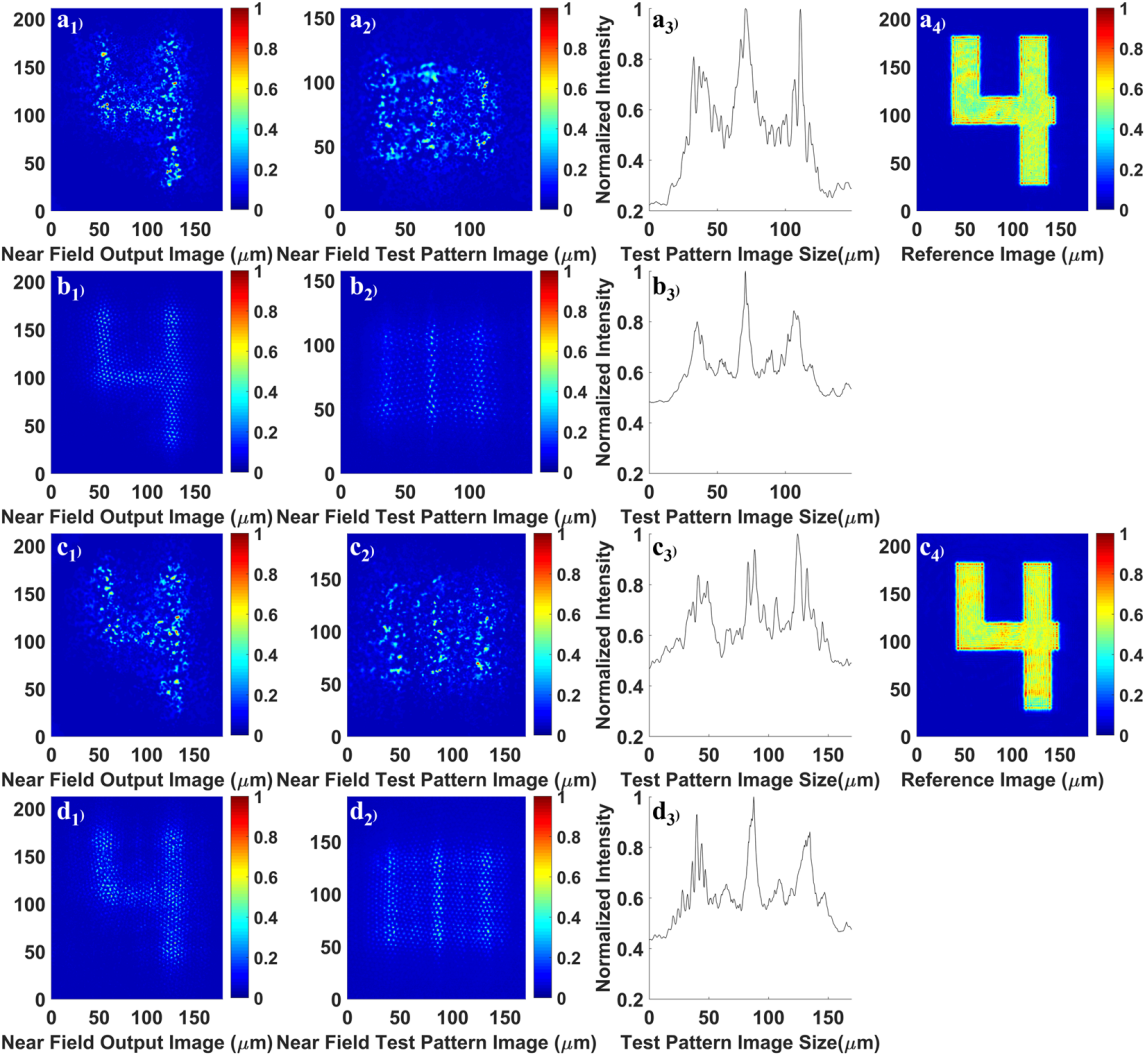
(**a1**–**a3**) and (**c1**–**c3**) are images of numbers and line elements and the corresponding intensity cross-sections of the line elements after transport through 4.5 cm of GARF(1). The integrated cross-sections are obtained by integration over the vertical axis of line elements. The reference images (**a4**) and (**c4**) are measured without any fiber; (**b1**–**b3**) and (**d1**–**d3**) are images of numbers and line elements and the corresponding intensity profiles of the line elements after transport through 4.5 cm of commercial imaging fiber FIGH-10–500N. The wavelength for a1)-a4) and b1)-b3) is 405 nm while the wavelength for (**c1**–**c4**) and (**d1**–**d3**) is 635 nm. The number “4” belongs to group 3 on the resolution test target. The line elements in (**a2**) and (**b2**) belong to group 4 number 6 on the resolution test target, and (**c2**) and (**d2**) belong to group 4 number 4. The visibility values (I_max_ − I_min_)/(I_max_ + I_min_) are 0.31 for (**a3**), 0.24 for (**b3**), 0.28 for (**c3**), and 0.28 for (**d3**), respectively. To calculate visibilities, the maximum values are calculated using the average of all three peaks and the minimum values are obtained using the average of the two valleys between the peaks.

**Figure 4:**
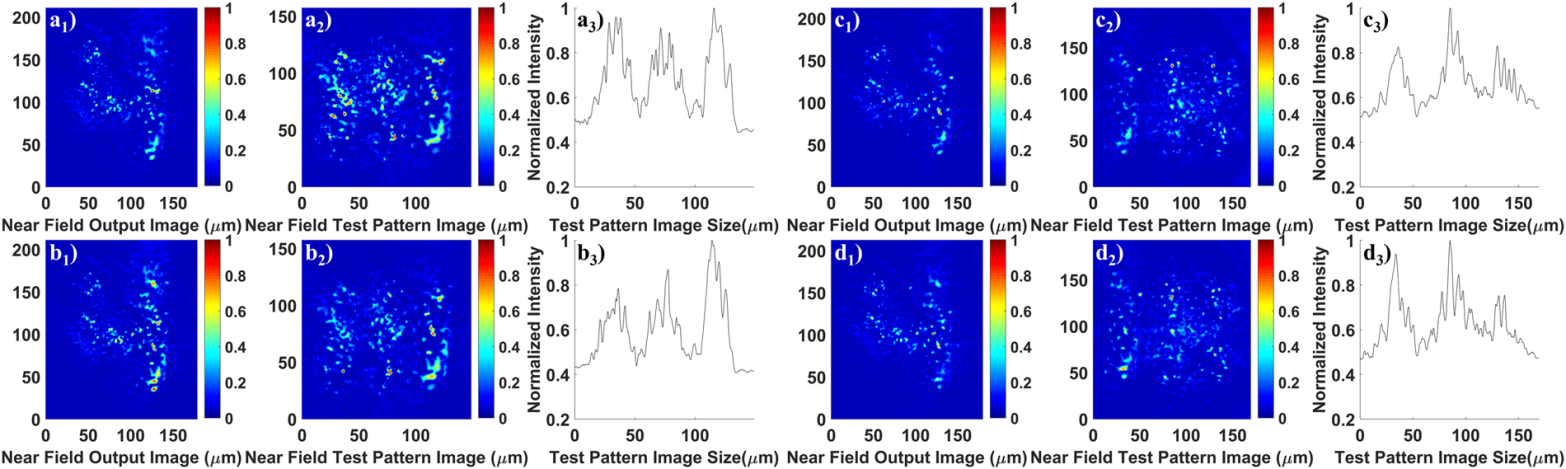
Images of numbers, line elements and the corresponding intensity profiles of the line elements after transport through 90 cm of GARF(1). (**a1**–**a3**) and (**c1**–**c3**) are the results obtained keeping the GARF(1) straight; while (**b1**–**b3**) and (**d1**–**d3**) are obtained using the same 90 cm long GARF(1) sample with a 180 degree turn (20 cm bending radius). The illumination wavelength for (a1–a3) and (b1–b3) is 405 nm, and the wavelength for (**c1**–**c3**) and (**d1–d3**) is 635 nm. The number “4” belongs to group 3 on the resolution target. The line elements in (**a2**) and (**b2**) belong to group 4 number 5 on the resolution test target. The line elements in (**c2**) and (**d2**) belong to group 4 number 3. The visibility values are 0.35 for (**a3**), 0.33 for (**b3**), 0.26 for (**c3**), and 0.31 for (**d3**), respectively.

**Figure 5:**
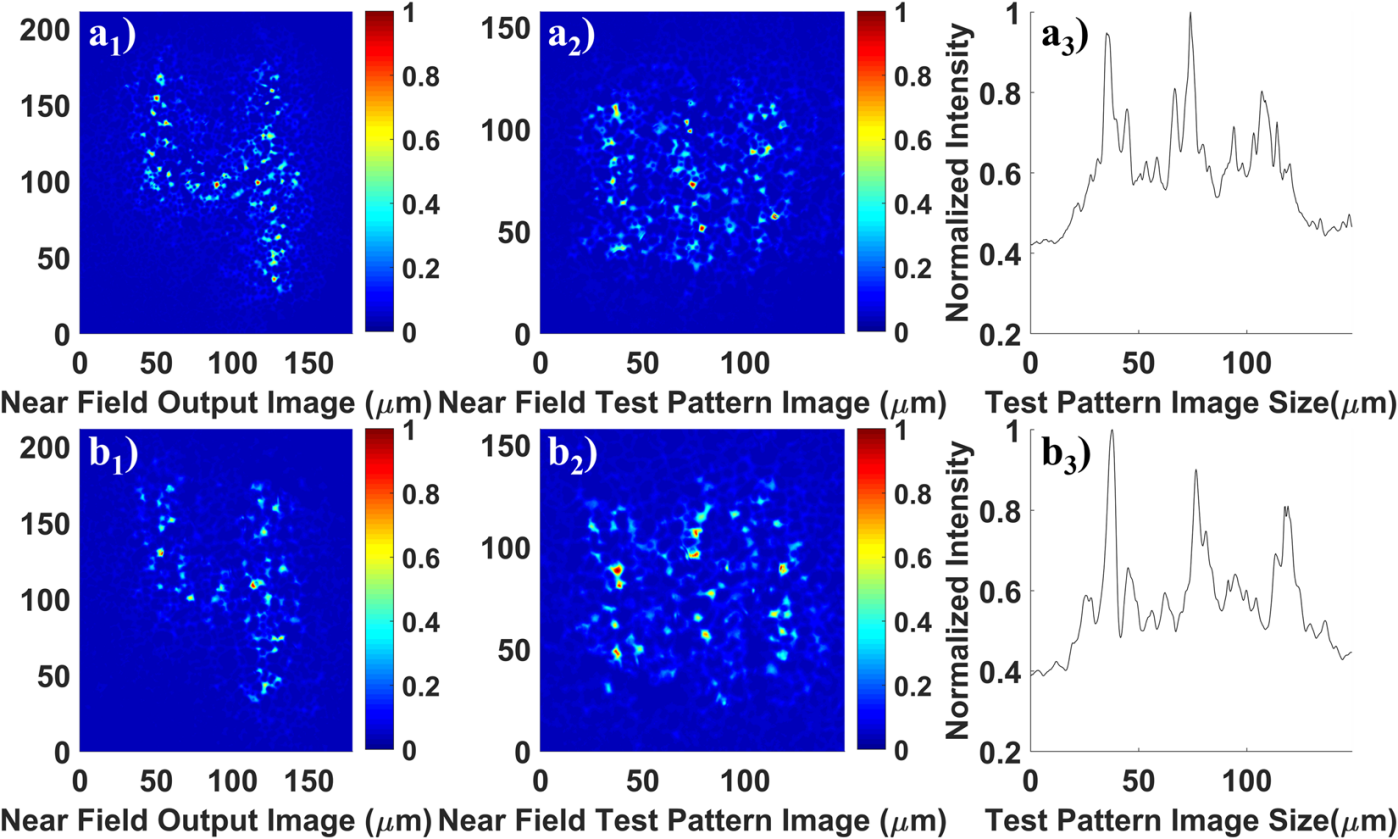
Images of numbers, line elements and the corresponding intensity cross-sections of the line elements after transport through 4.5 cm of the two different GARFs. (**a1**–**a3**) are obtained using a GARF(2) with a maximum of 6.8 μm^2^ in the air hole area distribution; (**b1**–**b3**) are obtained using a GARF(3) with a maximum of 18.5 μm^2^ in the air hole area distribution. The illumination wavelength is 405 nm. As in Figs 3 and 4, the number “4” belongs to group 3 on the resolution target. The line elements in (**a2**) belong to group 4 number 6 on the resolution target. The line elements in (**b2**) belong to group 4 number 5. The visibility values are 0.33 for (**a3**) and 0.32 for (**b3**).

For a more detailed evaluation, the imaging performance of the GARFs (see Fig. 1(a) has been analyzed quantitatively and compared with the commercially available Fujikura FIGH-10-500N multicore imaging fiber. To assess the transported image quality, we use the mean square error (MSE) and the mean structural similarity index (MSSIM) (see methods). The results are shown in Figs 3–5 and summarized in Table 1.

**Table 1:** MSE and MSSIM values of transported images.

Fiber	Fig. 3				Fig. 4				Fig. 5	
	a_1_	b_1_	c_1_	d_1_	a_1_	b_1_	c_1_	d_1_	a_1_	b_1_
	GARF(1)	FIGH	GARF(1)	FIGH	GARF(1)				GARF(2)	GARF(3)
Length	4.5 cm				90 cm				4.5 cm	
					straight	bend	straight	bend		
λ	405 nm		635 nm		405 nm		635 nm		405 nm	
MSE	0.049	0.053	0.055	0.056	0.053	0.051	0.060	0.060	0.051	0.054
MSSIM	0.317	0.315	0.252	0.237	0.246	0.306	0.206	0.215	0.263	0.241

Note that a smaller MSE value and a greater MSSIM value indicate higher fidelity to the original image. FIGH is the Fujikura FIGH-10-500N multicore imaging fiber.

The length of all fiber samples used in Fig. 3 is 4.5 cm, and the number “4” from group 3 in the resolution target has been transported. The wavelength used in Fig. 3(a1–a4) and 3(b1–b3) is 405  nm, while the wavelength used in Fig. 3(c1–c4) and (d1–d3) is 635 nm. Figure 3(a1–a3) and (c1–c3) are image transportation results utilizing the same GARF(1) sample; (b1–b3) and (d1–d3) are imaging results of the same FIGH-10–500N sample. The reference images used for the calculation of MSE and MSSIM values are shown in Fig. 3(a4) and (c4). These images were obtained without any fiber in the imaging set-up.

Table 1 summarizes all MSE and MSSIM values for images shown in Figs 3–5. Regarding Fig. 3, both MSE and MSSIM analysis indicate that the quality of images transported by GARF(1) is comparable to or even better than that of the images sent through the commercial multicore fiber FIGH-10–500N. In addition, the higher MSE values or lower MSSIM values at longer wavelength demonstrate that imaging quality decreases for both the GARF and the FIGH-10–500N with increasing wavelength. For the commercial multicore imaging fiber, the inter-core coupling strength increases with wavelength and, therefore, the image in Fig. 3(d1) appears slightly blurred. As for GARF(1), the image quality decreases since the mean transverse localized beam radius increases with wavelength, resulting in reduced resolution at longer wavelengths^10^.

To quantify the image resolution limit for transportation through the GARF and the FIGH-10–500N, images of 3-line elements on the resolution test target have been transported through both fibers. The smallest line elements that could be resolved for each sample were recorded with the CCD camera. As shown in Fig. 3(a2) and (b2), line elements of group 4 number 6 corresponding to a line width of 17.54 μm could be resolved after transportation through both fibers using 405 nm light. For the increased wavelength of 635 nm (see Fig. 3(c2) and (d2)), the smallest resolvable line elements belong to group 4 number 4 of the resolution target which have a line width of 22.10 μm. Without any fiber sample, the experimental setup can resolve the line elements of group 7 number 6. These are the smallest line elements of the 1951 USAF resolution test target with a line width of 2.19 μm. Therefore, the resolution of the transported images is indeed limited by the resolving power of the fiber; it is about the same for our GARF and the commercial fiber, and it is measured to be about 18 μm and 22 μm for 405 nm and 635 nm illumination, respectively.

### Image transport through meter-long straight and bent GARF

For practical applications that require flexible imaging fiber, the transport of high quality images through meter-long fiber, even with tight bends, is a key performance parameter. We have qualitatively demonstrated that our GARF can provide such characteristics, as shown in Fig. 2. We apply the MSE and MSSIM methods as the metric to quantify the image fidelity and measure the resolution limit for 90 cm long transport of images through GARF(1). Images of numbers and line elements that have been transported through the same 90 cm-long straight and bent GARF are shown in Fig. 4. The number “4” comes from group 3 on the resolution target. The illumination wavelength used in Fig. 4(a1–a3) and (b1–b3) is 405 nm, and the wavelength used in (c1–c3) and (d1–d3) is 635 nm. (a1–a3) and (c1–c3) are images through 90 cm of straight GARF(1); (b1–b3) and (d1–d3) are images through the same 90 cm-long GARF(1) sample with a 180 degree turn (20 cm bending radius). The MSE and MSSIM values of Fig. 4 are listed in Table 1, and these values compare well with those obtained through few cm-long GARF(1) samples shown in Fig. 3. In addition, comparing the MSE and MSSIM values obtained for the same wavelength, bending, at least to radii above 20 cm, does not result in a decrease of the quality of the transported images. The bending radius was limited to 20 cm to avoid breaking the GARF.

In addition, images of the smallest resolvable 3-line elements have also been taken to quantify the achievable spatial resolution. Figure 4(a2) and (b2) are images of line elements from group 4 number 5 of the resolution target with a line width of 19.69 μm, while Fig. 4(c2) and (d2) are images of line elements from group 4 number 3 with a line width of 24.8 μm. Based on these results, we can conclude that image transport through meter-long GARF is bending-independent for both image quality and spatial resolution, an observation that might be related to the single mode nature of the localized states in disordered fibers^11^. Due to the reasons mentioned above, both image quality and spatial resolution decrease slightly with increasing wavelength. Most importantly, image quality, spatial resolution and image brightness are only slightly lower when comparing transport through 90 cm and transport through 4.5 cm of GARF. These small degradations might be attributed to slight variation of fiber and feature dimensions along the light propagation direction due to fabrication imperfections.

### Impact of the feature size on image quality

So far, all the results are obtained using GARF(1) show in Fig. 1(a), which has a most common air hole area of about 2.5 μm^2^. To provide a first glance of the effect of varying the air hole distribution, we collected images transported through two other GARF samples with different maxima in the air hole area distributions but similar air filling fractions. These results are shown in Fig. 5.

The measurements shown in Fig. 5(a1–a3) are obtained using a GARF(2) sample with a maximum of 6.8 μm^2^ (Supplementary Fig. 1(b)) in the air hole area distribution, while a GARF(3) sample with a maximum of 18.5 μm^2^ (Supplementary Fig. 1(c)) in air hole area distribution is used to collect the data shown in (b1–b3). Again, the wavelength used for illumination was 405 nm, and the number “4” belongs to group 3 on the resolution target. The MSE and MSSIM values of Fig. 5 are listed in Table 1. The line elements of group 4 number 6 shown in Fig. 5(a2) have a line width of 17.54 μm and could be resolved after transportation through GARF(2). For transport through GARF(3), the smallest resolvable line elements (see Fig. 5(b2–b3)) belong to group 4 number 5 of the resolution target which have a line width of 19.69 μm.

Comparing the results of Figs 5 and 3(a1–a3), we observe that an increase in the maximum of the air hole area distribution from 2.5 μm^2^ to 6.8 μm^2^ and 18.5 μm^2^ led to a slight decrease in the image quality. However, the spatial resolution stays roughly the same for GARF(1) and GARF(2), while it slightly decreases for GARF(3) with the largest feature sizes. Although this is not at all a complete exploration of the possible parameter space for image transport in fibers with randomly disordered structures, we find that there is a general trend of a reduction in transported image quality and resolution when the air hole areas are increased within the range of investigation from about 2 μm^2^ to 20 μm^2^. This coincides with an observed increase of the localization length for larger air hole areas (see Supplementary Fig. 2).

Reasons for this trend include the fact that the mean-free path for wave scattering increases with feature size resulting in a larger localization radius, and, therefore, a degradation in image quality and resolution^6^. However, it should be noted that there are other factors influencing the image transport quality and resolution, for example, the air filling fraction and the width of the feature size distribution. While we demonstrated a trend, a complete optimization of the GARF design is beyond the scope of this paper.

## Conclusions

In conclusion, we have demonstrated the first silica-air randomly disordered fiber that can transmit high quality, high resolution images over meter scale distances. Quality and resolution compare well with one of the best commercial multicore imaging fibers. Bending the fiber to a radius of 20 cm does not noticeably influence the image transport performance making it suitable for applications where flexibility is required. In addition, we investigated the effects of wavelength variation and random feature sizes on the image quality experimentally, which could help to optimize the design of the next generation silica-air random fibers. Although the uniformity of the random structure, air filling fraction, and feature size distribution are still not perfect, the imaging transport demonstrated here paves the way for moving forward from proof-of-concept^5^ to performances suitable for applications.

## Methods

### MSE and MSSIM

To quantify the transported image quality, we use the mean square error (MSE) and the mean structural similarity index (MSSIM) throughout this manuscript. In both evaluations, images transported through the fiber are considered as distorted images, while images collected in the same setup without fiber are used as reference images (see Fig. 3(a4) and (c4)). MSE is the most widely used image quality measure, and is computed by averaging the squared error between the pixel intensities of distorted and reference images. MSE is calculated based on equation (1). X and Y are two 2D matrices that contain pixel intensities of distorted and reference images, respectively. M and N stand for the size of the matrices.

1 $$MSE=\frac{1}{M\times N}\sum _{i=1}^{N}\sum _{j=1}^{M}{({X}_{i.j}-{Y}_{i.j})}^{2}$$

MSSIM is another highly effective method for the assessment of image quality. It is calculated using methods introduced by Wang *et al*.^12,13^. The MSSIM index is calculated by averaging the structural similarity index (SSIM). SSIM is used to compare the local image patches from the same locations of the distorted image X and reference image Y. The SSIM index is calculated using equation (2).

2 $$SSIM(X,Y)=\frac{(2{\mu }_{X}{\mu }_{Y}+{C}_{1})(2{\sigma }_{XY}+{C}_{2})}{({\mu }_{X}^{2}+{\mu }_{Y}^{2}+{C}_{1})({\sigma }_{X}^{2}+{\sigma }_{Y}^{2}+{C}_{2})}$$

For image quality assessment, the SSIM index is applied locally rather than globally. μ_X_ and μ_Y_ are the local sample mean intensities of X and Y. σ_X_ and σ_Y_ are the local sample standard deviation of X and Y. σ_XY_ is the cross correlation of X and Y. C_1_ and C_2_ are used to avoid unstable results and defined as (K_1_L)^2^ and (K_2_L)^2^. L is the dynamic range of the pixel values of the image. K_1_ and K_2_ are two constants, and K_1_«1, K_2_«1. These constants are somewhat arbitrary and the SSIM index is fairly insensitive to the variation of their values. To evaluate the overall image quality, the MSSIM is calculated as equation (3)

3 $$MSSIM(X,Y)=\frac{1}{M}\sum _{j=1}^{M}SSIM({x}_{j},{y}_{j})$$

As mentioned before, X and Y are the distorted and the reference images, respectively. x_j_ and y_j_ are the image contents at the jth local window. M is the number of local windows of the image.

### Data availability

The datasets generated during the current study are available from the corresponding author under reasonable request.

## Electronic supplementary material

Supplementary Information

Supplementary Video
