# Supplementary material for: Image Transport Through Meter-Long Randomly Disordered Silica-Air Optical Fiber
Source: Sci Rep. 2018 Feb 15;8:3065. doi: 10.1038/s41598-018-21480-0 (PMC5814544; doi:10.1038/s41598-018-21480-0)
Supplement: Supplementary file 1 — Supplementary Information [file 41598_2018_21480_MOESM1_ESM.docx]

**Supplementary Information**

**Image Transport Through Meter-Long Randomly Disordered Silica-Air Optical Fiber**

**Jian Zhao^1*^, Jose Enrique Antonio Lopez^1^, Zheyuan Zhu^1^, Donghui Zheng^1,2^, Shuo Pang^1^, Rodrigo Amezcua Correa^1^ & Axel Schülzgen^1^**

^1^CREOL, College of Optics and Photonics, University of Central Florida, Orlando, FL 32816, USA

^2^School of Electronic and Optical Engineering, Nanjing University of Science and Technology, Nanjing, Jiangsu 210094, China

*Corresponding author: [JianZHAO@knights.ucf.edu](mailto:JianZHAO@knights.ucf.edu)

**Statistical distribution of air holes**


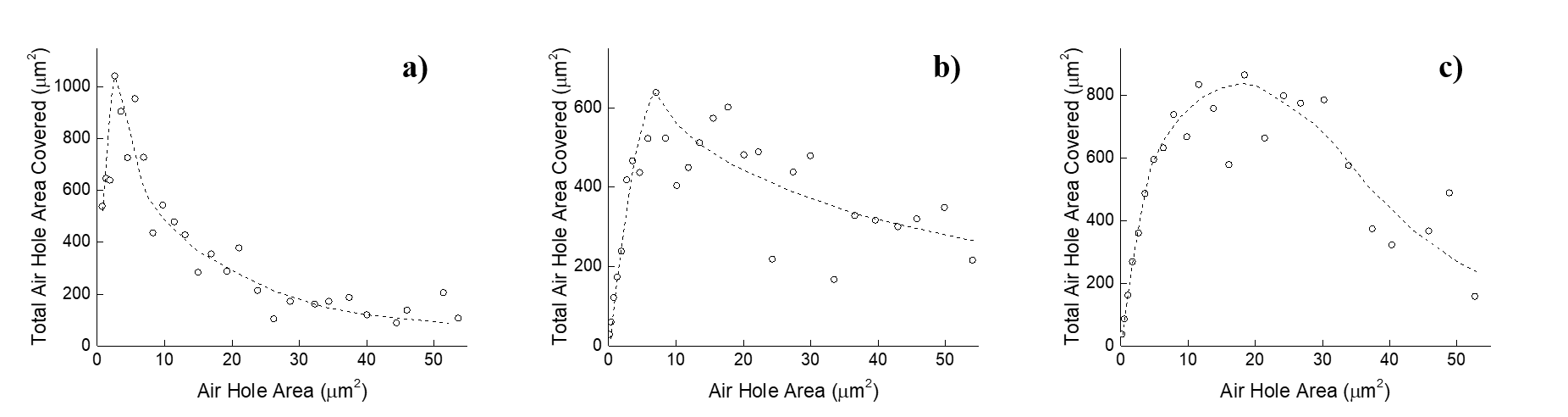


**Supplementary Figure 1.** Statistical distribution of air hole areas in the GARF samples. a) is GARF(1); b) is GARF(2); c) is GARF(3).

The air hole area distributions of GARF(1), GARF(2), and GARF(3) have maxima of 2.5 μm^2^, 6.8 μm^2^, and 18.5 μm^2^, respectively.

**Measurements of localization lengths**


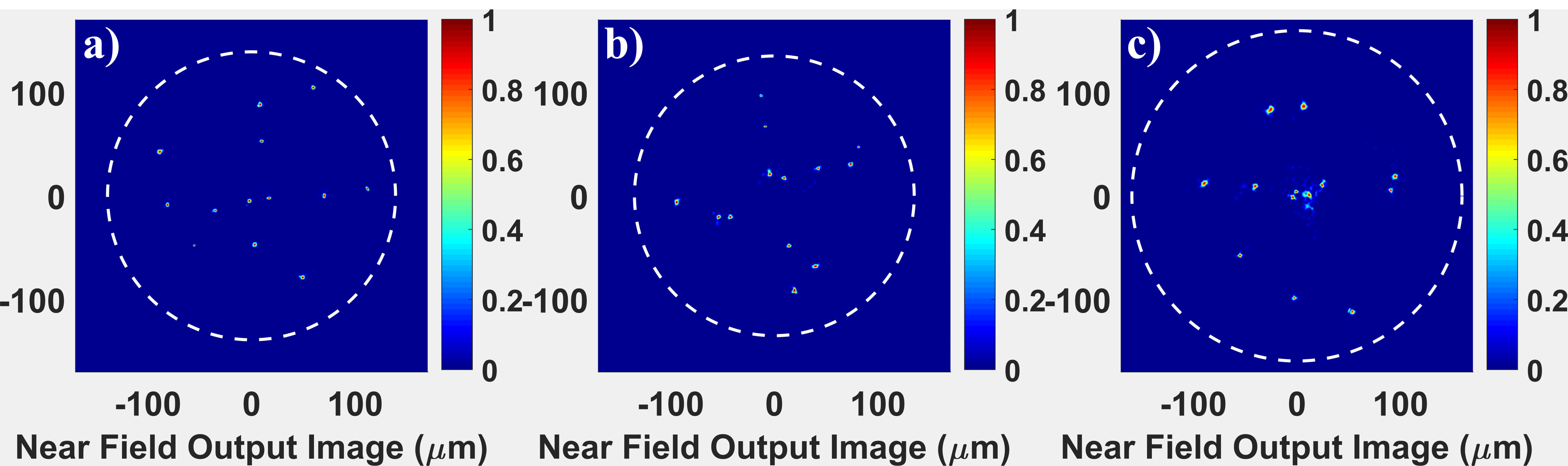


**Supplementary Figure 2.** Near field output images for multiple excitation positions recorded after transmission through a) GARF(1), b) GARF(2), and c) GARF(3), respectively. All fiber segments where 4.5 cm and the data were taken at a wavelength of 635 nm.

In analogy to reference [1], we measured the localization length of all three GARFs (4.5 cm long samples). A 635nm laser beam is delivered by single mode fiber (SMF), and is butt coupled to the GARF sample. The output facet of the GARF sample is imaged onto a CCD by a 20x objective. We locate the SMF at 13 different (x,y)-input positions of the disordered structures, and record 13 output beam profiles by a CCD camera for each GARF. In Fig. 2 (a) to (c) all 13 output beam profiles measured for each GARF are stacked on top of each other to create one single image per GARF. The white dashed line circles indicate the boundary of the disordered structures. For each recorded beam profile, we estimate the localization length using the following formula [2]:

$\frac{1}{L^{2}}=\frac{\int I(x.y)^{2}dxdy}{\left[\int I(x,y)dxdy\right]^{2}}$ (1)

where L is the localization length, and I(x,y) is the beam intensity at position (x,y). Then we obtain an estimate for the localization length of each GARF sample by averaging the 13 calculated values. Based on this method, the approximate localization lengths are 5.2 μm, 5.4 μm, and 6.8 μm for GARF(1), GARF(2), and GARF(3), respectively.

**References**

1. Karbasi, S., Mirr, C. R., Yarandi, P. C., Frazier, R. J., Koch, K. W., & Mafi, A. “Observation of transverse Anderson localization in an optical fiber,” Opt. Lett. **37**, 2304-2306 (2012).

2. Leonetti, M., Karbasi, S., Mafi, A., & Conti, C. Experimental observation of disordered induced self-focusing in optical fibers. Appl. Phys. Lett. **105**, 171102 (2014)
